# Effects of Licorice Stem and Leaf Forage on Growth and Physiology of Hotan Sheep

**DOI:** 10.3390/ani15101459

**Published:** 2025-05-18

**Authors:** Peng Yin, Weiqing Kong, Linyuan Cheng, Nana Shi, Shonghan Wang, Fei Guo, Haitao Shen, Hua Yao, Hongbin Li

**Affiliations:** 1 Key Laboratory of Xinjiang Phytomedicine Resource and Utilization, Ministry of Education, College of Life Sciences, Shihezi University, Shihezi 832003, China; yinyinan1010@163.com (P.Y.); kwq04200603@163.com (W.K.); c19109354382@163.com (L.C.); 13209988724@163.com (N.S.); 18540355042@163.com (S.W.); 13999416185@163.com (H.Y.); lihb@shzu.edu.cn (H.L.); 2 School of Life Sciences, Zhuhai College of Science and Technology, Zhuhai 519040, China; weisw2024@163.com; 3 Key Laboratory of Oasis Town and Mountain-Basin System Ecology of Xinjiang Production and Construction Corps, Shihezi University, Shihezi 832003, China

**Keywords:** licorice stems and leaves, meat sheep, growth performance, meat quality, immune performance, intestinal flora

## Abstract

This study explored the impact of licorice stem and leaf forage in the diet of meat sheep on their growth performance, physiological parameters, immunity, and gut microbiota. Conducted in the Hotan region of Xinjiang, the experiment involved 40 healthy local Hotan sheep (5–6 months old) that were randomly assigned to four groups, with both corn stalks and corn grain partially substituted at varying levels (30%, 40%, and 50%) by licorice stems and leaves for 80 days. Daily feed intake and refusal were recorded to assess feeding behavior and efficiency, while body weight was measured every 20 days to monitor growth performance. On day 80, blood samples were collected via venipuncture for biochemical and immune function analysis, followed by slaughter to obtain meat samples for quality assessment. Results showed that dietary licorice supplementation significantly improved growth performance. Sheep in the 40% replacement group had the highest final body weight and average daily gain (*p* < 0.05), indicating better feed efficiency. These findings suggest that replacing corn stalks and grain with licorice stems and leaves at a 40% ratio can significantly improve growth performance. Biochemical results showed improved protein metabolism and lipid profiles, with increased TP and ALB and decreased TC and TG levels. Immune indicators, including IgG, IgM, IgA, and IL-6, were also elevated in licorice-fed groups. In summary, licorice stems and leaves demonstrated clear nutritional and immunological benefits, especially at the 40% inclusion level. Gut microbiota analysis showed that licorice stems and leaves increased the relative abundance of Firmicutes while reducing Bacteroidota. At the genus level, UCG_005 and Bacteroides increased with higher licorice concentrations, whereas Treponema decreased. Pearson correlation analysis indicated that UCG_005 was positively correlated with TP, ALB, IgM, and IL-6 and negatively correlated with TG and TNF-α. These findings suggest that using licorice stems and leaves as a replacement for both corn stalks and corn grain modulates gut microbiota composition and is closely associated with immune and biochemical responses in sheep.

## Simple Summary

With increasing restrictions on antibiotics in livestock production, natural feed additives are gaining attention. Licorice stems and leaves, though often discarded, are rich in nutrients and bioactive compounds. This study evaluated their effects as a partial replacement for corn stalks as well as a portion of corn grain in sheep diets. Results showed that a 40% inclusion significantly improved growth, immunity, and gut health, supporting their sustainable use in animal nutrition.

## 1. Introduction

Licorice stems and leaves are agricultural by-products of the licorice root processing industry, primarily produced in China, Iran, and Central Asia. Global production of licorice root exceeds 120,000 tons annually. These residues are rich in flavonoids and polysaccharides, making them a promising sustainable feed resource. Utilizing such by-products aligns with global strategies for circular agriculture and waste reduction.

In livestock production, various medicinal plants are used as forage sources and feed additives to enhance animal performance [1,2,3,4,5,6,7]. Moreover, the use of agricultural by-products—for instance, ammoniated Cymbopogon nardus waste (ACNW)—was evaluated as a substitute for native grass in the diets of fattening Ettawa Crossbreed dairy goats. The study found that replacing native grass with ACNW did not result in any significant differences in nutrient intake, digestibility, or weight gain, indicating that ACNW is a viable alternative feed resource [8]. Licorice is a medicinal herb known for its root, which contains active compounds with various therapeutic properties [9]. However, its stems and leaves remain largely underutilized. These above-ground parts have considerable nutritional and medicinal potential that warrants further exploration [10]. Recent studies have shown that incorporating licorice stems and leaves into ruminant diets can improve animal performance and product quality. They are rich in nutrients, including proteins, carbohydrates, vitamins, essential and non-essential amino acids, and high levels of crude fiber, providing a nutritional profile superior to that of conventional straw-based roughages [11,12]. In addition, the unique flavor and aroma of licorice enhance feed palatability, stimulate appetite, and promote efficient digestion and nutrient absorption [13,14,15].

Beyond their nutritional value, licorice stems and leaves contain various active ingredients, such as glycyrrhizic acid, saponins, flavonoids, and polysaccharides [16], which exhibit significant antioxidant activity [17], antioxidant and anti-inflammatory effects [18], and immunomodulatory effects [19]. Studies have shown that these components can effectively enhance immune function, reduce morbidity, and improve disease resistance in animals. For instance, licorice supplementation improved immunity and reduced disease incidence in rabbits and enhanced immune responses and health status in beef cattle, thereby providing a natural alternative to antibiotics in animal production. [20,21]. With the growing challenge of antibiotic resistance, the unique advantages of licorice stems and leaves as natural forage are becoming increasingly prominent, offering both improved animal health and a sustainable solution for the livestock industry [22,23,24,25,26].

In recent years, it has been increasingly recognized that the gut microbiota plays a critical role in animal health and production performance. The balance of gut flora affects animals’ digestive and absorptive capacity and is closely related to their immune function, metabolic regulation, and disease resistance. Wang et al. reported that traditional Chinese medicine can effectively increase the abundance of short-chain fatty acid-producing beneficial bacteria, reduce the abundance of pathogenic bacteria, restore the balance of intestinal flora, indirectly alleviate the dysfunction of the intestinal mucosal immune barrier, and promote the repair of damaged intestinal mucosa [27]. It has also been shown that the growth performance, activity of non-specific immune parameters, immune gene expression, and resistance to bacterial pathogens in grouper were significantly improved by modulating the intestinal flora and serum metabolic profiles after supplementation with Chinese herbs [28]. By adjusting the replacement ratio of licorice stems and leaves, it is possible to optimize the intestinal microenvironment of sheep, enhance the digestion and absorption efficiency of nutrients, and further improve their growth rate and meat quality. Particularly in the context of the restricted use of antibiotics, it is of great application value to explore the modulatory effects of natural feed additives on gut microorganisms, especially their effects on improving host health and production performance.

In this study, we aimed to investigate the effects of the inclusion proportion of licorice stems and leaves in the feed of fattening sheep on their immune function, growth performance, meat quality, and intestinal microbiota. Our findings will help establish an optimal feeding strategy and provide a foundational basis for the subsequent development of licorice-based feed for use in sheep husbandry. This research is significant for elucidating the nutritional value and efficacy of licorice stems and leaves, offering theoretical support for their application in veterinary nutrition. Moreover, optimizing the use of licorice stems and leaves in feed can effectively enhance sheep growth and health, presenting an economical, efficient, and sustainable feed ingredient for the livestock industry, thereby promoting the green development of the entire sector.

## 2. Materials and Methods

### 2.1. Animal Ethics Statement

The present study followed the recommendations of the Care and Use of Laboratory Animals in China, via the Animal Ethical and Welfare Committee of China Experimental Animal Society. The trial was approved by the Institutional Animal Care and Use Committee of Shihezi University (Xinjiang Uygur Autonomous Region, China) under protocol code A2023-287, and the trial procedures were conducted in accordance with Chinese animal welfare guidelines.

### 2.2. Experimental Design and Animal Management

The licorice used was Glycyrrhiza uralensis, cultivated in the experimental fields of Minfeng County Industrial Park. The licorice stems and leaves were manually harvested at the optimal growth stage in early autumn.

The study was conducted in the Hotan region of Xinjiang, with 40 male Hotan sheep aged 5 to 6 months randomly selected to ensure uniformity across experimental groups. The sheep had an average weight of 22.20 kg, with a standard deviation of 1.48 kg, and were healthy and well nourished. They were randomly divided into four groups of 10 animals each, with each group housed separately. In the Glyc30 group, 30% of the combined corn stalk and corn grain components of the basal diet were replaced with licorice stems and leaves; likewise, the Glyc40 and Glyc50 groups received 40% and 50% replacements, respectively. Thus, sheep in the Glyc30, Glyc40, and Glyc50 groups were identified as such, while the control group received the unmodified basal diet.

In the feed, licorice stems and leaves and corn stalks were sun-dried, chopped into small pieces, and included in the daily ration at 30%, 40%, or 50% inclusion levels (corresponding to approximately 450 g, 600 g, and 750 g per day, as fed, respectively) alongside the basal diet. All animals were fed using a total mixed ration (TMR), meaning that both concentrate and roughage were mixed together, with a total daily feed amount of 1500 g/day. All experimental groups were fed four isoenergetic and isonitrogenous diets to ensure that any differences in performance were solely due to the inclusion proportion of licorice.

To maintain optimal animal health and promote growth, the sheep pen featured a natural earthen floor. The breeding facility underwent thorough disinfection prior to the experiment, aimed at preventing disease transmission, enhancing hygiene, and improving feed quality in the housing environment. Disinfection measures included the application of chemical agents to the ground surfaces, water troughs, and equipment. Regular removal of organic materials, such as feces and leftover feed, was also implemented, along with diligent monitoring and maintenance to ensure ongoing cleanliness. The sheep pen was disinfected every 10 days. The sheep were fed twice daily at 08:00 and 18:00. Feed was provided ad libitum, with approximately 10% refusal allowed to ensure constant access to feed. Feed amounts were adjusted based on the actual intake levels, as determined through periodic observations of feeding behavior and activity. Periodic inspections were conducted to monitor the sheeps’ feeding behavior and activity. Additionally, rams were surgically castrated at least three weeks before the start of the experiment to ensure hormonal stabilization, reduce reproductive capacity and aggressive behavior, and simplify management.

The basal diets were formulated based on NRC [29] standards, specifically designed for castrated male sheep with a body weight of approximately 22 kg and an expected daily gain of 200 g/day. The analyzed nutrient levels of these diets are presented in Table 1. The ingredient composition of the basal diet is detailed in Table 2. Conventional nutrients, including crude protein (CP) and crude fat (EE), were analyzed following the methods outlined by AOAC [30]. Acid detergent fiber (ADF) and neutral detergent fiber (NDF) were determined using the technique described by Van Soest et al. [31], while total calcium (Ca) and total phosphorus (P) were analyzed according to the method described by Hambleton et al. [32]. The sheep in the above experimental groups had the same water and management except for the variables set.

### 2.3. Weight Gain Performance

On the 0th, 20th, 40th, 60th, and 80th day of the experiment, each group of experimental sheep was weighed on an empty stomach and recorded, and the average daily gain (ADG) was calculated. The feed intake and residual feed of each group were accurately recorded once daily at 08:00 (prior to administering the morning feed), and the average daily feed intake (ADFI, dry matter, g/day) was calculated accordingly. The feed-to-gain ratio (F/G) was calculated based on the average daily feed intake (dry matter, g/day) and average daily gain.

### 2.4. Blood Indicators

On day 80, 5 mL of blood was collected from the jugular vein using a disposable syringe and transferred into a sterilized centrifuge tube. The blood samples were centrifuged at 3500 rpm for 15 min at 4 °C to separate the serum. Total protein (TP), albumin (ALB), alanine aminotransferase (AST), aspartate aminotransferase (ALT), total cholesterol (TC), triglycerides (TGs), and blood urea nitrogen (BUN) were measured according to the instructions provided with the blood biochemical index kits. These biochemical parameters were determined using the Beckman AU680 Fully Automatic Biochemical Analyzer (Beckman, Brea, CA, USA). Immunity indicators, including immunoglobulin M (IgM), immunoglobulin G (IgG), immunoglobulin A (IgA), interleukin-2 (IL-2), interleukin-6 (IL-6), and tumor necrosis factor-alpha (TNF-α), were assessed following the protocols outlined in the immunity indicator kits. These immune parameters were measured using the Roche 601 Electrochemiluminescence Fully Automatic Immunoassay Analyzer.

### 2.5. Meat Quality

On day 80, after slaughter, samples of the longissimus dorsi muscle were collected from both sides at the 12th–13th thoracic vertebrae. One side was used for pH measurement and color analysis, while the other side was used for water loss evaluation and cookability assessment. Immediately after slaughter, muscle samples were carefully excised, trimmed of visible fat and connective tissue, and cut into uniform sections for subsequent analysis. Muscle pH was measured using a solid-state pH meter (Testo 205, Testo SE, Titisee-Neustadt, Germany) inserted into the longissimus dorsi muscle at three different locations per sample, and the average value was recorded [33]. The measurement was repeated after 24 h of storage at 4 °C. Meat color parameters, including lightness (L*), redness (a*), and yellowness (b*), were assessed 45 min post-slaughter using a portable colorimeter (CR-400, Konica Minolta Sensing Inc., Ramsey, NJ, USA) following the guidelines of the American Meat Science Association (AMSA, 2012) [34]. Each sample was measured three times and averaged. Drip loss was calculated as the percentage weight loss after suspending the sample in a sealed container at 4 °C for 24 h [35]. Cooking loss was assessed by measuring the weight difference before and after heating the sample in a 75 °C water bath for 30 min. Shear force was determined using a texture analyzer (TA.XT Plus, Stable Micro Systems Ltd., Godalming, UK), with the probe cutting perpendicular to the muscle fibers of the cooked samples, as described by Honikel (1998) [36].

### 2.6. Characterization of Intestinal Flora

On day 80, samples of approximately 2–5 g of fresh sheep feces were collected and placed into sterile containers for subsequent 16S rRNA gene sequencing analysis. To ensure sample integrity, all specimens were immediately frozen and transported to the laboratory on sufficient dry ice. Upon arrival, the samples underwent quality assessment and processing. DNA was extracted, and its quality was evaluated based on concentration and purity, typically using a spectrophotometer or bioanalyzer.

The raw sequencing data have been deposited in the NCBI Sequence Read Archive (SRA) under the accession number PRJNA1252279. For the subsequent data analysis, software packages such as QIIME2 (v2023.2), Mothur (v1.48.0), or other relevant bioinformatics tools were utilized to process the sequencing data. These tools facilitated the identification of various microbial taxa and allowed for downstream statistical analyses to better interpret the microbial community structure present in the fecal samples.

### 2.7. Data Processing

Excel was initially used to process the experimental data, followed by statistical analysis using one-way ANOVA in SPSS 17.0. GraphPad Prism 8.0 was utilized to generate the corresponding plots. Gut microbiota data were analyzed and visualized using QIIME2 (v2023.2) and R software (v4.3.1) (including the phyloseq and ggplot2 packages). The results are presented as mean ± standard error of the mean (SEM), with a *p*-value < 0.05 indicating statistical significance and *p* < 0.01 denoting high significance.

## 3. Results

### 3.1. Weight Gain Performance

As shown in Table 3, dietary replacement of corn stalks as well as a portion of corn grain with licorice stems and leaves significantly affected the FBW and ADG of sheep (*p* < 0.001). The Glyc40 group achieved the highest FBW (33.08 kg) and ADG (123.34 g/day), both significantly greater than the control and other treatment groups (*p* < 0.05). The Glyc30 and Glyc50 replacement groups also showed improved FBW and ADG compared to the control group, although values were lower than those of Glyc40.

ADFI and F/G were recorded at the group level and are, therefore, presented descriptively without statistical comparison. ADFI tended to increase in all licorice-supplemented groups relative to the control. The lowest F/G (11.54) was observed in the Glyc40 group, indicating higher feed efficiency at this replacement level.

### 3.2. Effects of Blood Biochemical Indicators

As shown in Table 4, there were no significant differences in ALT and AST among the control and licorice-supplemented groups (*p* > 0.05), indicating that licorice did not adversely affect liver function. Similarly, BUN did not differ among groups (*p* > 0.05), suggesting that kidney function remained unaffected. By contrast, TP and ALB were significantly higher in the Glyc40 group than in the control group (*p* < 0.001), reflecting enhanced protein metabolism. Additionally, TC was significantly lower in all licorice-supplemented groups compared to the control (*p* < 0.001). TG levels were also lower in the licorice-fed groups (*p* < 0.001). Overall, these results suggest that an appropriate amount of licorice stems and leaves can improve protein synthesis and regulate lipid metabolism without negatively impacting liver or kidney health.

### 3.3. Effects of Blood Immunological Indicators

As shown in Table 5, replacing corn stalks as well as a portion of corn grain with licorice stems and leaves significantly affected immune function in sheep. IgM, IgG, and IgA levels were significantly increased in the licorice groups (*p* < 0.05), with the highest IgA observed in Glyc50. IL-6 was significantly higher in the Glyc40 group than in the control and Glyc50 groups (*p* < 0.05). No significant differences were found in IL-2 and TNF-α levels among groups. These results suggest that licorice supplementation enhances immune function.

### 3.4. Meat Quality

As shown in Table 6, replacing corn stalks as well as a portion of corn grain with licorice stems and leaves did not have a statistically significant effect on meat quality in sheep, such as the pH at 45 min (*p* = 0.322), pH at 24 h (*p* = 0.301), Loss of water/% (*p* = 0.897), Cooked meat rate/% (*p* = 0.644), Tenderness (*p* = 0.563), Color-Brightness (*p* = 0.145), Color-Redness (*p* = 0.607), and Color-Yellowness (*p* = 0.201).

### 3.5. Intestinal Flora

#### 3.5.1. Alpha Diversity and Beta Diversity Analysis

**Figure 1:**
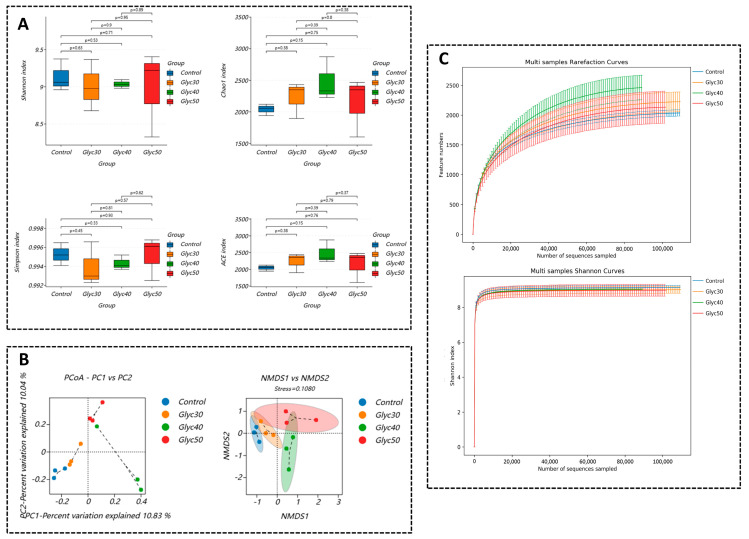
Feasibility analysis of Alpha Diversity and Beta Diversity OUTs. (**A**) Chao1, Ace, Shannon, Simpson, Chao1, and Ace indices measure species richness, i.e., the number of species. In contrast, Shannon and Simpson’s indices measure species diversity, which is influenced by species richness and community evenness in the sample community. For the same species richness, the greater evenness each species in the community has, the greater diversity the community is considered to have. The greater the value of the Shannon index and the greater the value of the Simpson index, the greater the species diversity of the sample; (**B**) Non-Metric Multi-Dimensional Scaling (NMDS) is a method for measuring species diversity in a sample community. NMDS is a sorting method applicable to ecological research, mainly a data analysis method that simplifies research objects (samples or variables) in multi-dimensional to low-dimensional space for locating, analyzing, and categorizing while preserving the original relationship between objects; (**C**) shows an index reflecting the diversity of microorganisms in a sample, using the microbial Shannon index constructed from the sequencing volume of each sample at different sequencing depths.

Alpha diversity analysis: As shown in Figure 1A, the richness (Shannon, Simpson, Chao1, ACE indices) of each group was not significantly different (*p* > 0.05). In addition, the Shannon index curve and the sparsity curve tended to be flat, indicating that the amount of sequencing data was large enough and that the amount of sequencing data of the samples was reasonable (Figure 1C).

The NMDS plot is a sorting method applicable to ecological research, an important indicator reflecting the degree of difference between samples. When the stress value is less than 0.1, it is considered a good ordination; when stress is less than 0.05, the representation is deemed very good. Generally, a stress value less than 0.2 indicates that the NMDS analysis has a certain level of reliability. In the coordinate plot, samples that are closer together exhibit greater similarity. The results of this study show that the stress value is 0.1080, which indicates a good degree of variation between groups. PCoA plotting is a technique for analyzing and simplifying data sets by decomposing the variance and reflecting the differences between multiple data groups on a two-dimensional coordinate plot. The closer the two samples are, the more similar the composition of these two samples is. As shown in Figure 1B, the flora of the control group was similar to that of the Glyc30 group, while it was relatively distant and discrete in distribution from the Glyc40 and Glyc50 groups. Meanwhile, the Glyc30 group, Glyc40 group, and Glyc50 group were close.

#### 3.5.2. Species Taxonomy and Correlation Analysis

**Figure 2:**
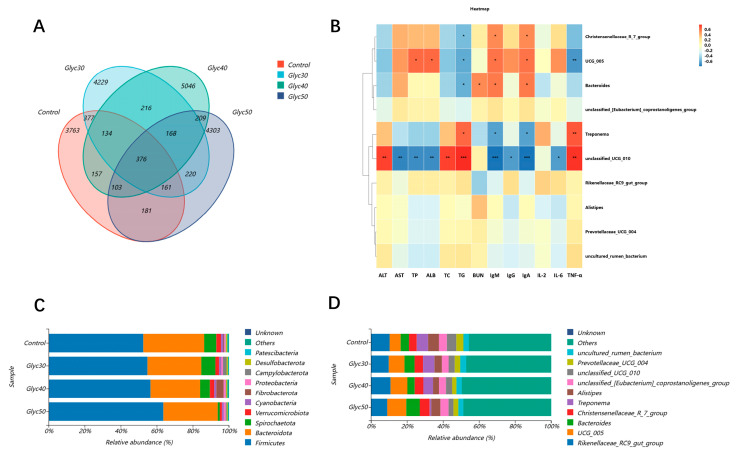
Taxonomic analysis of species. (**A**) Each circle of the Venn diagram represents a subgroup; the overlapping part is the number of species shared among the samples, and the non-overlapping part is the number of species-specific to the subgroup; (**B**) shows the results of the correlation analysis of the immunological and biochemical indicators with the intestinal microbiota by Pearson’s rank correlation coefficients at the genus level. The color corresponds to the legend: red is a positive correlation, blue is a negative correlation, and the darker the color, the higher the correlation; * in the figure indicates a significant correlation (* <0.05, ** <0.001, *** <0.0001); (**C**) relative abundances at the level of bacterial phyla; (**D**) relative abundances at the level of bacterial genera.

As shown in Figure 2A, the total number of operational taxonomic units (OTUs) in the control group sample was 5252, with 3763 unique OTUs. In the 30% licorice group, the total OTUs were 5881, comprising 4229 unique OTUs. The Glyc40 group exhibited 6409 total OTUs and 5046 unique OTUs, while the Glyc50 group had 5721 total OTUs and 4303 unique OTUs. Across all groups, the number of OTUs was consistent at 376.

As shown in Figure 2C, the results indicate that the intestinal flora of sheep was predominantly composed of four species: Firmicutes, Bacteroidota, Spirochaetota, and Verrucomicrobiota, which collectively accounted for over 90% of the community. Within the control group, Firmicutes represented 52.46% of the relative abundance, while in the Glyc30, Glyc40, and Glyc50 groups, it increased to 60.34%, 56.90%, and 60.30%, respectively. Conversely, the relative abundance of Bacteroidota decreased slightly from 33.77% in the control group to 27.95%, 30.19%, and 31.40% in the experimental groups, respectively. The relative abundance of Spirochaetota was 6.75% in the control group but dropped to 5.43% and 1.18% in the Glyc40 and Glyc50 groups, respectively. Verrucomicrobiota accounted for 2.61% in both the control and Glyc30 groups, while in the Glyc40 and Glyc50 groups, it decreased to 1.55% and 1.20%, respectively.

As shown in Figure 2D, at the genus level, Rikenellaceae_RC9_gut_group represented 10.28% in the control group, with slightly reduced abundances of 9.78%, 10.79%, and 8.94% observed in the Glyc30, Glyc40, and Glyc50 groups, respectively. The abundance of UCG_005 increased with higher licorice concentrations, accounting for 6.09% in the control group and rising to 8.70%, 9.30%, and 10.58% in the Glyc30, Glyc40, and Glyc50 groups, respectively. Bacteroides increased in abundance from 4.64% in the control group to 5.10%, 6.14%, and 7.47% in the experimental groups, respectively. Meanwhile, the abundance of Treponema was 6.28% in the control group but significantly decreased to 5.82%, 4.47%, and 0.93% in the Glyc30, Glyc40, and Glyc50 groups, respectively. Alistipes decreased slightly from 6.04% in the control group to 5.09% in the Glyc50 group. In contrast, the unclassified Eubacterium coprostanoligenes group remained relatively stable at 4.40% in the control group and slightly increased to 4.49% in the Glyc50 group. The relative abundance of Prevotellaceae UCG_004 decreased from 3.94% in the control group to 2.75% in the Glyc50 group. The abundance of unknown taxa remained very low across all groups, at 0.00% or 0.01%.

As shown in Figure 2B, Pearson’s rank correlation coefficient analysis revealed notable correlations between microbial composition and immune and biochemical parameters. The Christensenellaceae R_7_group demonstrated a positive correlation with IgM and IgA while showing a negative correlation with triglycerides (TGs). The unclassified_005 group was positively correlated with total protein (TP), albumin (ALB), IgM, and interleukin-6 (IL-6) and negatively correlated with TGs and tumor necrosis factor-alpha (TNF-α). Bacteroides exhibited positive correlations with blood urea nitrogen (BUN), IgM, and IgA but negatively correlated with TGs. Treponema showed positive correlations with TGs and TNF- α and negative correlations with IgM and IgA. Finally, unclassified_UCG_010 was positively correlated with ALT, total cholesterol (TC), TGs, and TNF-α while demonstrating negative correlations with AST, TP, ALB, IgM, IgG, IgA, and IL-6.

#### 3.5.3. Differability Analysis Between Groups

**Figure 3:**
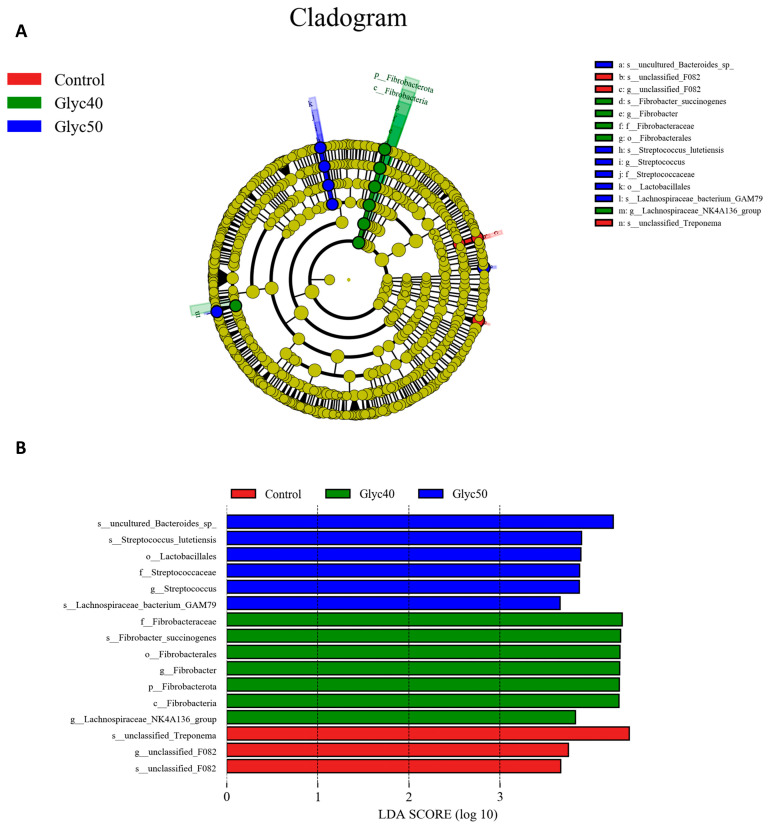
Linear discriminant analysis coupled with effect size (LEFse). (**A**) Cladogram. The circle radiates inside out, demonstrating the classification—from the kingdom to species. Each small circle at different classification represents a taxon and the diameter of the circle is proportional to the relative abundance. Species not with significant differences are colored yellow, and biomarker groups are colored differently. Red and green dots represent the core bacterial populations in the respective group. Abbreviation: LDA, linear discriminant analysis. (**B**) The histogram of the LDA scores. The degrees of influence of species are expressed by the lengths of the bars in the histogram.

To identify bacterial taxa that were significantly differentiated between the groups, a linear discriminant effect size (LEfSe) analysis (LDA score > 3.5) was performed (Figure 3). The s_unclassified_Treponema, g_unclassified_F082, and s_unclassified_F082 were more abundant in the control group; f_Fibrobacteraceae, s_Fibrobacter_succinogenes, o_Fibrobacterales, g_Fibrobacter, p_Fibrobacterota, c_Fibrobacteria, and g_Lachnospiraceae_NK4A136_group are more abundant in the Glyc40 group; s_uncultured_Bacteroides_sp, s_Streptococcus_lutetiensis, o_Lactobacillales, f_Streptococcaceae, g_Streptococcus, and s_Lachnospiraceae_bacterium_GAM79 were more abundant in the Glyc50 group. However, there was no significant difference in microbiota abundance between the Glyc30 and control groups.

## 4. Discussion

This study demonstrated that incorporating licorice stems and leaves into the diet enhanced growth performance in mutton sheep, with the Glyc40 group showing the highest final body weight and average daily gain (ADG). Although average daily feed intake (ADFI) and feed conversion ratio (F/G) were measured at the group level and, thus, not subjected to statistical analysis, a consistent trend toward improved feed utilization was observed in the licorice-supplemented groups. As all diets were formulated to be isonitrogenous and isoenergetic, the enhanced performance—particularly in the 40% licorice group—is likely attributable to the bioactive compounds in licorice stems and leaves. These compounds, including flavonoids, saponins, and glycyrrhizin, are known to stimulate digestive enzyme activity, improve gut function, and enhance nutrient absorption. Together, these effects likely contributed synergistically to the observed improvements in growth rate and feed efficiency. Similar results were reported by Naseri et al. [37], where licorice extract improved the growth performance, rumen fermentation parameters, and protozoa populations in fattening sheep, further supporting the positive role of licorice-derived feed components in ruminant nutrition.

Serum biochemical indicators are often used as one of the most important indicators to evaluate an animal’s health. In the present study, liver function indicators such as ALT and AST were maintained in the normal range (*p* > 0.05), indicating that licorice did not adversely affect the liver. At the same time, blood urea nitrogen (BUN) did not change significantly in all groups (*p* > 0.05), suggesting that it did not hurt renal function. Serum total protein (TP) and albumin (ALB) can reflect the body’s status in protein synthesis and metabolism and, to a certain extent, also reflect the body’s ability to digest and utilize proteins and its immune level [38]. Serum total protein (TP) and albumin (ALB) are key markers of protein synthesis, nutritional status, and immune competence. In this experiment, TP and ALB levels were significantly increased in the licorice-fed groups (*p* < 0.05), especially in the Glyc40 group, reflecting improved protein metabolism and potential immunonutritional benefits. These findings are consistent with previous studies showing that licorice extract improved serum protein levels and liver enzyme profiles in broilers and sheep [39,40].

In terms of lipid metabolism, licorice supplementation significantly reduced serum total cholesterol (TC) and triglyceride (TG) levels (*p* < 0.05), indicating its lipotropic effects. Similar lipid-lowering effects of licorice have been observed in broiler chickens [41] and beef cattle [21], suggesting its potential role in regulating fat metabolism and improving overall metabolic health in livestock.

Licorice stems and leaves has been shown to effectively enhance livestock immune function. Previous studies have reported that supplementing 1000 mg/kg of licorice polysaccharides significantly increased serum IgG, IgM, and total antioxidant capacity in weaned piglets (*p* < 0.05) [42]. Similarly, in quail studies, both the licorice polysaccharide and whole licorice supplementation groups exhibited significantly higher IgG and IgM levels than the control group on day 50 (*p* < 0.05) [43].Consistent with these findings, our study demonstrated that serum IgA, IgG, and IgM levels were significantly higher in the experimental groups receiving different proportions of licorice stems and leaves compared to the control group. IL-6 is a pleiotropic cytokine produced by multiple cell types—including macrophages and lymphocytes—that plays a key role in acute inflammatory responses. IL-6 promotes B-cell differentiation and antibody production and helps regulate both immunity and inflammation in vivo [44]. In our trial, IL-6 concentrations were significantly higher in the Glyc40 group than in the control group (*p* < 0.05). This elevation of IL-6 may reflect enhanced macrophage activation and cytokine secretion induced by licorice bioactives, suggesting that licorice supplementation not only boosts antibody production but also promotes a more robust acute-phase immune response. These results further confirm that licorice stems and leaves can effectively enhance immune function in livestock, reinforcing their potential as a natural immunomodulatory feed additive.

Regarding the gut microbiota, the alpha diversity analysis in this study showed no significant differences between groups. Two possible factors may account for this discrepancy. First, the gut microbial ecosystem of sheep is relatively stable, making it less susceptible to disturbances from a single plant-derived compound. Second, this study directly incorporated licorice stems and leaves rather than refined licorice extract, potentially leading to differences in the concentration and release rate of active compounds, resulting in only subtle adjustments to microbial abundance and composition. Although no significant changes were observed in alpha diversity, beta diversity analysis revealed notable shifts in microbial community structure. PCoA and NMDS plots showed that the microbiota of the control group was closely related to that of the Glyc30 group, whereas the Glyc40 and Glyc50 groups exhibited a more dispersed and distinct community distribution. This suggests that as the proportion of licorice supplementation increased, the gut microbiota of sheep underwent significant structural reorganization—indicating changes in species composition, even though the overall microbial diversity remained largely unchanged.

Further phylum-level analysis revealed that licorice supplementation significantly increased the relative abundance of Firmicutes while markedly decreasing Verrucomicrobiota. Firmicutes are closely linked to the production of short-chain fatty acids (SCFAs), particularly butyrate, which is vital for enhancing host insulin sensitivity and exerting anti-inflammatory effects [45]. Meanwhile, Bacteroidota escapes proximal digestion of carbohydrates and indigestible oligosaccharides through fermentation, which in turn synthesizes butyrate, propionate, and acetate, among others, and these short-chain fatty acids are considered to be a rich source of energy for their hosts [46]. It was reported that when the ratio of Bacteroidota to Bacteroidota was elevated, it helped to promote obesity in mice [47]. These findings suggest that bioactive components in licorice stems and leaves, such as polysaccharides and saponins, could modulate the gut microenvironment, thereby optimizing the growth of beneficial bacteria and enhancing nutrient absorption and immune regulation.

At the genus level, we found that the abundance of Bacteroides and UCG_005 increased with increasing licorice concentration, while Treponema decreased significantly. Bacteroides plays a key role in resolving complex polysaccharides, promoting nutrient absorption, and maintaining intestinal barrier integrity [48]. Studies have shown that Bacteroides intervention, which reduces the disruption of gut flora induced by lipopolysaccharide treatment and maintains the integrity of the intestinal epithelium and plasma lipopolysaccharide concentrations, can promote intestinal homeostasis [49]. There are also studies showing that Bacteroides promotes IgM serum antibody concentrations [50]; this is consistent with our findings on blood markers. UCG_005 is a potentially beneficial bacterium, and some antioxidants, such as chlorogenic acid, have been shown to increase the abundance of UCG_005 while increasing the antioxidant status of broiler chickens [51]. Treponema is a genus of anaerobic, spiral-shaped, and highly motile bacteria strongly associated with various animal diseases [52]. For example, Treponema has also been implicated in diseases such as bovine dermatitis, which affects the animals’ physiological state and causes economic losses to the farming industry [53]. Prevotellaceae UCG_004, belonging to the Prevotellaceae family of bacteria, helps to enhance rumen fermentation and rumen epithelial development in animals [54]. These bacteria are described in detail and specialize in polysaccharide degradation and production of acetate and butyrate [55,56]. Similarly, Li et al. [57] found that Prevotellaceae UCG_004 can ferment carbohydrates and produce SCFA, including acetate and butyrate. The significant reduction of Prevotellaceae UCG_004 in our experiment in high concentrations of licorice stem and leaf feeding and its growth performance was not as expected, which may be related to this bacterium. There is some correlation between these microbiota changes and our observed serum immune indicators (e.g., IgA and IgM levels), suggesting that licorice stem and leaf supplements play a dual role in improving immune function and metabolism in sheep by regulating intestinal microflora structure.

This study utilized Pearson correlation coefficients to investigate the relationship between microbial community composition and immune and biochemical indicators. The analysis results indicate that different microbial communities play significant roles in regulating immune functions and metabolic pathways. Notably, the Christensenellaceae R_7_group exhibited a positive correlation with immunoglobulin M (IgM) and immunoglobulin A (IgA), while showing a negative correlation with triglycerides (TG). The Christensenellaceae R_7_group is an important member of the Christensenellaceae family, which plays a crucial role in amino acid and lipid metabolism [58]. Christensenellaceae is closely associated with mammalian health, exhibiting an inverse relationship with fat content; higher relative abundance of this family is beneficial for health [59]. This is consistent with our results, suggesting that this group of bacteria may play a role in modulating immune responses and enhancing immune function, while potentially promoting cardiovascular health by lowering blood lipid levels.

Additionally, bacteria of the genus Bacteroides have shown a positive correlation with blood urea nitrogen (BUN), immunoglobulin M (IgM), and immunoglobulin A (IgA), while exhibiting a negative correlation with triglycerides (TGs). Bacteroides species are primarily involved in the digestion of cellulose, and their high abundance in the gut environment contributes to meeting the nutritional and energy needs of animals during growth and development [60]. Bacteroides contains a substantial number of Gram-positive bacteria, some of which are considered beneficial, as they help resist the invasion of pathogenic microorganisms and maintain intestinal microbial balance [61]. This suggests that Bacteroides may play a significant role in improving protein metabolism, enhancing immune function, and regulating lipid metabolism.

Additionally, Treponema shows a positive correlation with triglycerides (TGs) and tumor necrosis factor-alpha (TNF-α), while exhibiting a negative correlation with immunoglobulin M (IgM) and immunoglobulin A (IgA). The genus Treponema comprises anaerobic, spiral-shaped, and highly motile bacteria, including various pathogenic and symbiotic species that inhabit distinctly different anatomical and environmental niches in both humans and animals [62]. Treponema is associated with colitis, including the invasion of surface epithelium and the superficial layers of the mucosa [63]. Research has found that Treponema promotes the development of immune thrombocytopenic purpura (ITP) through the mediation of vascular endothelial growth factor A (VEGF-A) [64]. In this study, feeding licorice stems and leaves successfully reduced the abundance of Treponema in the gut microbiota of meat sheep and significantly increased the levels of IgM and IgA in the blood. These results suggest that modulating the gut microbiota, particularly by decreasing levels of the harmful bacterium Treponema, may provide an effective strategy for improving immune function and preventing related diseases.

Finally, unclassified_UCG_010 showed a positive correlation with several physiological indicators, including ALT, total cholesterol (TC), triglycerides (TGs), and TNF-α, while exhibiting a negative correlation with AST, total protein (TP), albumin (ALB), IgM, IgG, IgA, and IL-6. In contrast, the UCG_005 group demonstrated an entirely opposite effect on physiological indicators as compared to unclassified_UCG_010. Previous research has indicated that the UCG_005 of Ruminococcus provides benefits to the host by preventing diabetes and enhancing levels of short-chain fatty acids (SCFAs) in the gut [65]. Research has found that the abundance of the family UCG_005 of Ruminococcus is significantly reduced in the intestines of diarrheic goat kids compared to healthy goat kids [66]. In this study, licorice stems and leaves increased the abundance of the family UCG_005 of Ruminococcus while decreasing the abundance of unclassified_UCG_010. This phenomenon suggests that by modulating the gut microbiota structure and increasing the proportion of beneficial bacteria, it is possible to not only improve metabolic health but also potentially enhance immune function. This underscores the potential application value of licorice stems and leaves in regulating gut microbial composition.

In summary, the findings of this study provide strong evidence for a deeper understanding of the multifaceted roles played by the gut microbiota in immune regulation and metabolic processes.

In exploring the effects of different licorice replacement ratios on sheep meat quality, although some literature [67,68,69] reported that plant extracts could significantly improve meat quality, results in this study, however, did not find that the replacement of licorice stems and leaves significantly improved the texture and color indexes of sheep meat. This may be due to the complex mechanism of the active ingredient in licorice in meat formation, or the feeding cycle, addition amount, and interaction factors, which should be further explored in subsequent studies.

In conclusion, licorice stems and leaves as feed not only significantly improved the growth performance index of sheep, but also improved the immune and metabolic status by regulating the structure of gut microbiota.

## 5. Conclusions

Replacing corn stalks as well as a portion of corn grain and leaves in the diet of meat sheep effectively enhances growth performance and feed efficiency. The most notable improvements were observed at 30% and 40% inclusion levels, with the 40% group achieving the highest final body weight and average daily gain. Although the 50% replacement also outperformed the control group, its effects were less pronounced. These improvements are likely attributable to the bioactive compounds present in licorice stems and leaves—such as flavonoids and saponins—which are known to enhance digestive enzyme activity, support gut health, and promote nutrient absorption. While feed intake and feed conversion ratio were not statistically analyzed due to group-level data collection, the overall trends support enhanced feed utilization. Additionally, licorice supplementation positively influenced immune responses and modulated the gut microbiota. Future studies should incorporate individual-level intake data and detailed digestibility trials to clarify the underlying mechanisms and determine optimal inclusion levels for sustainable and efficient sheep production.

## Figures and Tables

**Table 1:** Chemical composition of individual ingredients (air-dried basis, %).

Ingredient	Crude Protein	Crude Fat	Acid Detergent Fiber	Neutral Detergent Fiber	Total Calcium	Total Phosphorus
**Licorice stems and leaves**	12.37	1.95	35.12	60.47	0.81	0.16
**Corn stalks**	3.52	1.48	39.88	75.05	0.47	0.12
**Corn**	8.12	4.05	4.75	11.87	0.02	0.34

**Table 2:** Composition and nutrient levels of diets (air-dried basis, %).

Item	Diet			
	Control	Glyc30	Glyc40	Glyc50
Ingredients				
**Licorice**	0	30.00	40.00	50.00
**Corn stalks**	60.00	39.00	34.00	30.00
**Corn**	25.00	16.00	11.00	5.00
**Bran**	2.00	7.50	8.50	10.00
**Soybean meal**	8.00	2.00	1.00	1
**Cotton meal**	2.00	2.5	2.50	1
**Premix** ^1^	1.00	1.00	1.00	1.00
**Edible salt**	1.00	1.00	1.00	1.00
**Baking soda**	1.00	1.00	1.00	1.00
**Total**	100.00	100.00	100.00	100.00
**Nutrient levels** ^2^				
**Crude protein**	11.53	11.84	11.47	11.56
**Crude fat**	2.56	2.39	2.32	2.37
**Acid detergent fiber**	23.79	24.40	26.09	24.90
**Neutral detergent fiber**	50.29	47.57	45.32	48.71
**Total calcium**	1.29	1.23	1.24	1.24
**Total phosphorus**	0.24	0.21	0.21	0.22

Notes: ^1^ Premix composition (per kg): VE 213 KIU, VA 404 KIU, VD3 11 KIU, Cu 400 mg, Fe 1320 mg, Zn 1400 mg, Mn 760 mg. ^2^ Nutrient levels are measured values.

**Table 3:** Effects of different licorice replacement ratios on growth performance of sheep.

Treatment									
Items	Control	Glyc30	Glyc40	Glyc50	SEM	*p*-Value	P (Linear)	P (Quadratic)	P (Cubic)
**IBW, kg**	22.80	22.55	23.21	23.12	0.27	0.708	0.415	0.512	0.826
**FBW, kg**	26.28 ^c^	30.94 ^b^	33.08 ^a^	29.60 ^b^	0.53	<0.001	<0.001	0.014	0.465
**1–80 d**									
**ADG (g/day)**	43.51 ^d^	104.87 ^b^	123.34 ^a^	81.04 ^c^	2.32	<0.001	<0.001	<0.001	0.065
**ADFI** ^1^ **(g/day)**	1261.37	1417.75	1423.68	1404.25					
**F/G** ^1^	28.99	13.52	11.54	17.33					

Notes: The experimental groups were fed licorice stem and leaf instead of corn stalk, with replacement proportions of 30% (Glyc30), 40% (Glyc40), and 50% (Glyc50) of the total diet, while the control group was fed a basal diet. a, b, c, d mean that values within a row with different superscript letters were significantly different. *p* < 0.05 is considered statistically significant. *p*-value: the significance of the difference among treatments. IBW: initial body weight, FBW: final body weight, ADG: average daily gain, ADFI: average daily feed intake, F/G: feed-to-gain ratio. ^1^ Feed intake was measured at the group (pen) level; therefore, average daily feed intake (ADFI) and feed-to-gain ratio (F/G) are presented as descriptive values only and were not subjected to statistical analysis.

**Table 4:** Effects of different licorice replacement ratios on blood biochemical indexes of sheep.

Items	Control	Glyc30	Glyc40	Glyc50	SEM	*p*-Value	P (Linear)	P (Quadratic)	P (Cubic)
**ALT (U/L)**	17.16	16.39	17.05	16.51	0.31	0.756	0.043	0.122	0.581
**AST (U/L)**	106.98	113	109.92	112.47	1.701	0.592	0.223	0.951	0.662
**TP (g/L)**	53.24 ^c^	58.33 ^b^	64.1 ^a^	61.98 ^ab^	0.817	<0.001	<0.001	0.015	0.347
**ALB (g/L)**	23.31 ^c^	25.44 ^b^	28.57 ^a^	27.13 ^a^	0.369	<0.001	0.032	<0.001	0.235
**TC (mmol/L)**	2.05 ^a^	1.25 ^b^	1.52 ^b^	1.42 ^b^	0.072	<0.001	<0.001	0.026	0.481
**TG (mmol/L)**	1.60 ^a^	0.86 ^b^	0.90 ^b^	0.83 ^b^	0.538	<0.001	0.063	0.154	0.826
**BUN (mmol/L)**	3.55	3.54	3.42	3.76	0.061	0.240	0.759	0.423	0.687

Notes: The experimental groups were fed licorice stem and leaf instead of corn stalk, with replacement proportions of 30% (Glyc30), 40% (Glyc40), and 50% (Glyc50) of the total diet, while the control group was fed a basal diet. a, b, c, mean that values within a row with different superscript letters were significantly different. *p* < 0.05 is considered statistically significant. *p*-value: the significance of the difference among treatments. ALT: Alanine aminotransferase, AST: Aspartate aminotransferase, TP: Total Protein, ALB: Albumin, TC: Cholesterol, TG: Triglyceride, BUN: Blood Urea Nitrogen.

**Table 5:** Effects of different licorice replacement ratios on blood immunity indexes of sheep.

Items	Control	Glyc30	Glyc40	Glyc50	SEM	*p*-Value	P (Linear)	P (Quadratic)	P (Cubic)
**IgM (g/L)**	21.61 ^b^	23.52 ^a^	23.00 ^ab^	23.72 ^a^	0.265	0.016	0.004	0.021	0.367
**IgG (g/L)**	21.10 ^b^	26.37 ^a^	26.48 ^a^	25.98 ^a^	0.448	<0.001	<0.001	0.052	0.187
**IgA (g/L)**	9.85 ^d^	17.26 ^b^	15.98 ^c^	19.22 ^a^	0.542	<0.001	0.002	0.011	0.312
**IL-2 (pg/mL)**	852.67	911.04	855.90	841.12	12.05	0.169	0.154	0.547	0.772
**IL-6 (pg/mL)**	66.77 ^b^	75.54 ^ab^	86.93 ^a^	72.45 ^b^	2.110	0.004	0.045	0.236	0.639
**TNF-α (pg/mL)**	294.37	293.58	282.94	280.67	6.066	0.806	0.112	0.422	0.698

Notes: The experimental groups were fed licorice stem and leaf instead of corn stalk, with replacement proportions of 30% (Glyc30), 40% (Glyc40), and 50% (Glyc50) of the total diet, while the control group was fed a basal diet. a, b, c, d mean that values within a row with different superscript letters were significantly different. *p* < 0.05 is considered statistically significant. *p*-value: the significance of the difference among treatments. IgM: Immunoglobulin M, IgG: Immunoglobulin G, IgA: Immunoglobulin A, IL-2: Interleukin-2, IL-6: Interleukin-6, TNF-α: Tumor necrosis factor-α.

**Table 6:** Effects of different licorice replacement ratios on meat quality of sheep.

Items	Control	Glyc30	Glyc40	Glyc50	SEM	*p*-Value	P (Linear)	P (Quadratic)	P (Cubic)
**pH at 45 min**	6.21	6.21	6.23	6.20	0.003	0.322	0.416	0.281	0.725
**pH at 24 h**	6.03	5.99	5.98	5.97	0.001	0.301	0.122	0.198	0.554
**Loss water rate (%)**	19.11	20.07	19.95	19.98	0.079	0.897	0.477	0.614	0.832
**Cooking loss rate (%)**	75.03	75.11	75.21	75.26	0.246	0.644	0.204	0.327	0.757
**Tenderness (N)**	8.12	8.11	8.23	8.16	0.032	0.563	0.384	0.192	0.669
**Color-Brightness**	48.02	48.07	48.16	48.10	0.099	0.145	0.268	0.354	0.781
**Color-Redness**	13.78	14.08	13.91	13.84	0.118	0.607	0.355	0.421	0.912
**Color-Yellowness**	6.97	7.01	7.03	7.01	0.021	0.201	0.171	0.263	0.689

Notes: The experimental groups were fed licorice stem and leaf instead of corn stalk, with replacement proportions of 30% (Glyc30), 40% (Glyc40), and 50% (Glyc50) of the total diet, while the control group was fed a basal diet. *p* < 0.05 is considered statistically significant. *p*-value: the significance of the difference among treatments.

## Data Availability

The original contributions presented in this study are included in the article. Further inquiries can be directed to the corresponding author.

## Abbreviations

ADG	Average Daily Gain
ADFI	Average Daily Feed Intake
TP	Total Protein
ALB	Albumin
AST	Aspartate aminotransferase
ALT	Alanine aminotransferase
TG	Triglyceride
TC	Cholesterol
BUN	Blood Urea Nitrogen
IgM	Immunoglobulin M
IgA	Immunoglobulin G
IgG	Immunoglobulin G
IL-2	Interleukin-2
IL-6	Interleukin-6
TNF-α	Tumor necrosis factor-α

## References

[1] Maña M.A.T., Niepes R.A., Florida M.A.C., Paculba R.A. (2023). Growth performance of goats (*Capra hircus* L.) on forage legumes mixed with guinea grass (*Megathyrsus maximus*). J. Glob. Innov. Agric. Sci..

[2] Hegazy S.A., Abd E.S., Khorshed M., Salem F. (2023). Productive and immunological performance of small ruminants offered some medicinal plants as feed additives. Int. J. Vet. Sci..

[3] Baz M.M., Alfagham A.T., Al-Shuraym L.A., Moharam A.F. (2024). Efficacy and Comparative Toxicity of Phytochemical Compounds Extracted from Aromatic Perennial Trees and Herbs against Vector Borne Culex pipiens (Diptera: Culicidae) and *Hyalomma dromedarii* (Acari: Ixodidae) as Green Insecticides. Pak. Vet. J..

[4] Al-Hoshani N., Almahallawi R., Al-Nabati E.A., Althubyani S.A., Negm S., El-lkott A.F., Bajaber M.A., Soliman S.M., Ahmed A.E. (2024). Anthelmintic effects of herbal mixture of selected plants of *Apiaceae* on *Strongylus vulgaris* and *Fasciola hepatica*. Pak. Vet. J..

[5] Abdallah A., Zhang P., Elemba E., Zhong Q., Sun Z. (2020). Carcass characteristics, meat quality, and functional compound deposition in sheep fed diets supplemented with *Astragalus membranaceus* by-product. Anim. Feed Sci. Technol..

[6] Ardani L., Zain M., Elihasridas E., Pazla R., Utami Y., Sari R., Hadiwijaya M., Putri E., Makmur M. (2024). Optimization Indigofera zollingeriana and Gambier (*Uncaria gambir*) supplementation on feed consumption, digestibility, methane production and lactation performance of Etawa crossbreed goats. Int. J. Vet. Sci..

[7] Mohammad L.M., Kamil A.M., Tawfeeq R.K., Ahmed S.a.J. (2023). Ameliorating effects of herbal mixture for dexamethasone induced histological changes in mice. Int. J. Vet. Sci..

[8] Ningrat R.W.S., Zain M., Elihasridas E., Negara W., Masdia E., Putri R.P., Shafura P.O., Amanah U. (2024). The Effect of Ammoniated Cymbopogon nardus Waste as Forage Substitution on Nutrient Digestibility and Performance of Ettawa Crossbreed Dairy Goat. Int. J. Vet. Sci..

[9] Mamedov N.A., Egamberdieva D. (2019). Phytochemical constituents and pharmacological effects of licorice: A review. Plant and Human Health, Volume 3: Pharmacology and Therapeutic Uses.

[10] Esmaeili H., Mirjalili M.H., Zandi F. (2022). Antimicrobial and antioxidant activities of the leaf extract of some cultivated Iranian licorice populations. J. Med. Plants.

[11] Chen F., Wang J., Zhang S., Chaudhry A.S., Khanaki H. (2024). Assessing Fermentation Quality, Aerobic Stability, In Vitro Digestibility, and Rumen Degradation Characteristics of Silages Mixed with Sweet Sorghum and Aerial Parts of Licorice. Agriculture.

[12] Pan J., Dilibaier A., Xie Y.Y. (2019). Nutritional value and utilization of *Glycyrrhiza uralensis* stems and leaves in ruminants feed. CABI Digit. Libr..

[13] Zhao Y., Li C., Wang X., Wang Z., Wang J., Zhen W., Huang S., Li T., Fan H., Ma Y. (2023). Effects of *Glycyrrhiza polysaccharide* on growth performance, appetite, and hypothalamic inflammation in broilers. J. Anim. Sci..

[14] Wang Y., Xu W., Zhang J., Liu J., Wang Z., Liu Y., Mai K., Ai Q. (2022). Effects of Glycyrrhizin (GL) Supplementation on Survival, Growth Performance, Expression of Feeding-Related Genes, Activities of Digestive Enzymes, Antioxidant Capacity, and Expression of Inflammatory Factors in Large Yellow Croaker (*Larimichthys crocea*) Larvae. Aquac. Nutr..

[15] Cooney A.S., Fitzsimons J.T. (1996). Increased sodium appetite and thirst in rat induced by the ingredients of liquorice, glycyrrhizic acid and glycyrrhetinic acid. Regul. Pept..

[16] Ji X., Liu N., Huang S., Zhang C. (2024). A comprehensive review of licorice: The preparation, chemical composition, bioactivities and its applications. Am. J. Chin. Med..

[17] Petrosyan H.R., Nigaryan A.A., Hovhannisyan H.A., Soloyan A.M., Vardapetyan V.V., Martiryan A.I. (2023). Evaluation of antioxidant activity and heavy metals content in licorice (*Glycyrrhiza glabra* L.) growing wild in Armenia. Heliyon.

[18] Eltahir A.O.E., Lategan K.L., David O.M., Pool E.J., Luckay R.C., Hussein A.A. (2024). Green Synthesis of Gold Nanoparticles Using Liquiritin and Other Phenolics from *Glycyrrhiza glabra* and Their Anti-Inflammatory Activity. J. Funct. Biomater..

[19] Zendejas-Hernandez U., Alcántara-Martínez N., Vivar D.T., Valenzuela F., Sosa Espinoza A., Cervera Ceballos E.E. (2023). Nebulized glycyrrhizin/enoxolone drug modulates IL-17A in COVID-19 patients: A randomized clinical trial. Front. Immunol..

[20] Meng X.-l., You F., Cao H., Cai H.-m., Li Y., Yang G.-k., Zhang Y.-m., Chang X.-l., Zhang X.-d., Tian X. (2022). Effects of dietary licorice (*Glycyrrhiza uralensis*) supplementation on growth performance, muscle quality, and immunity in the common carp (*Cyprinus carpio haematopterus*). Aquac. Rep..

[21] Liang S., Meng J., Tang Z., Xie X., Tian M., Ma X., Yang X., Xiao D., Wang S. (2024). Licorice Extract Supplementation Benefits Growth Performance, Blood Biochemistry and Hormones, Immune Antioxidant Status, Hindgut Fecal Microbial Community, and Metabolism in Beef Cattle. Vet. Sci..

[22] Ramesh K.S.V., Gokul M.N.R., Penmetsa G.S., Sruthima G., Mohan Kumar P., Swetha P., Vivek B. (2024). Quantitative determination of the antibacterial activity of licorice (*Glycyrrhiza glabra*) and tetracycline gel against *Aggregatibacter actinomycetemcomitans* (Aa), *Porphyromonas gingivalis* (Pg) and *Prevotella intermedia* (Pi)—A microbiological in vitro study. J. Complement. Integr. Med..

[23] Gomaa A.A., Abdel-Wadood Y.A. (2021). The potential of glycyrrhizin and licorice extract in combating COVID-19 and associated conditions. Phytomedicine Plus.

[24] Yang J., Zhang L., He X., Gou X., Zong Z., Luo Y. (2024). In vitro and in vivo enhancement effect of glabridin on the antibacterial activity of colistin, against multidrug resistant Escherichia coli strains. Phytomedicine.

[25] Li Z., Dong M., Chen Z., Zhang C., Jiang J., Liu M., Cui Q. (2024). Combining virus-based affinity ultrafiltration method with serum pharmacochemistry to identify the antiviral pharmacodynamic substances in licorice. J. Ethnopharmacol..

[26] Bai W., Zhu Q., Wang J., Jiang L., Guo D., Li C., Xing X., Sun D. (2024). Licorice extract inhibits porcine epidemic diarrhea virus in vitro and in vivo. J. Gen. Virol..

[27] Wang M., Fu R., Xu D., Chen Y., Yue S., Zhang S., Tang Y. (2024). Traditional Chinese Medicine: A promising strategy to regulate the imbalance of bacterial flora, impaired intestinal barrier and immune function attributed to ulcerative colitis through intestinal microecology. J. Ethnopharmacol..

[28] Yang Y., Wang Y., Zhao L., Wang F., Li M., Wang Q., Luo H., Zhao Q., Zeng J., Zhao Y. (2023). Chinese herbal medicines for treating ulcerative colitis via regulating gut microbiota-intestinal immunity axis. Chin. Herb. Med..

[29] Council N.R. (2007). Nutrient Requirements of Small Ruminants: Sheep, Goats, Cervids, and New World Camelids.

[30] Horwitz W. (2000). Official Methods of Analysis of the Association of Official Analytical Chemists.

[31] Van Soest P.v., Robertson J.B., Lewis B.A. (1991). Methods for dietary fiber, neutral detergent fiber, and nonstarch polysaccharides in relation to animal nutrition. J. Dairy Sci..

[32] Hambleton L.G. (1977). Semiautomated method for simultaneous determination of phosphorus, calcium, and crude protein in animal feeds. J. Assoc. Off. Anal. Chem..

[33] Horwitz W., Albert R., Deutsch M.J., Thompson N.J. (1990). Precision parameters of methods of analysis required for nutrition labeling. Part I. Major nutrients. J. Assoc. Off. Anal. Chem..

[34] American Meat Science Association (2012). AMSA Meat Color Measurement Guidelines: AMSA[M].

[35] Cetin O., Bingol E.B., Colak H., Hampikyan H. (2012). Effects of electrical stimulation on meat quality of lamb and goat meat. Sci. World J..

[36] Honikel K.O. (1998). Reference methods for the assessment of physical characteristics of meat. Meat Sci..

[37] Naseri Moghadam A., Nooriyan Soroor M.E., Hozhabri F. (2023). The effect of licorice extract on growth performance of fattening lambs, fermentation parameters and rumen protozoan population. J. Anim. Prod..

[38] Song K., Shan A., Li J. (2004). Effect of different combinations of enzyme preparation supplemented to wheat based diets on growth and serum biochemical values of broiler chickens. Acta Zoonutrimenta Sin..

[39] Rezaei M., Kalantar M., Nasr J. (2014). *Thymus vulgaris* L., *Glycyrrhiza glabra*, and combo enzyme in corn or barley-basal diets in broiler chickens. Int. J. Plant Anim. Environ. Sci..

[40] Abo-Samaha M.I., Alghamdi Y.S., El-Shobokshy S.A., Albogami S., El-Maksoud E.M.A., Farrag F., Soliman M.M., Shukry M., El-Hack M.E.A. (2022). Licorice extract supplementation affects antioxidant activity, growth-related genes, lipid metabolism, and immune markers in broiler chickens. Life.

[41] Dheyauldeen Salahdin O., Othman H., Hafsan H., Mohammed F., Ahmed Hamza T., Kadhim M.M., Aravindhan S., Prakaash A.S., Fakri Mustafa Y. (2023). Effect of Licorice Essential Oil (*Glycyrrhizaglabraglabra*) on Performance and Some Biochemical Parameters of Broiler Chickens. Arch. Razi Inst..

[42] Li C., Zhao P., Shao Q., Chen W., Huang S., Wang X., Zhang C., He L. (2023). Effects of dietary *Glycyrrhiza polysaccharide* on growth performance, blood parameters and immunity in weaned piglets. J. Anim. Physiol. Anim. Nutr..

[43] Zhang C., Li C., Shao Q., Wang X., Chen W., Li Y., Huang S., Ma Y. (2023). Effects of dietary *Glycyrrhiza polysaccharide* on growth, serum biochemistry, immunity, and egg laying in quail. Anim. Biotechnol..

[44] Zhang W., Zheng H. (2017). Research progress of IL-6-mediated immune inflammatory response and its relationship with disease. Chin. J. Cell. Mol. Immunol..

[45] Magne F., Gotteland M., Gauthier L., Zazueta A., Pesoa S., Navarrete P., Balamurugan R. (2020). The firmicutes/bacteroidetes ratio: A relevant marker of gut dysbiosis in obese patients?. Nutrients.

[46] Jandhyala S.M., Talukdar R., Subramanyam C., Vuyyuru H., Sasikala M., Nageshwar Reddy D. (2015). Role of the normal gut microbiota. World J. Gastroenterol..

[47] Grigor’eva I.N. (2021). Gallstone Disease, Obesity and the firmicutes/bacteroidetes ratio as a possible biomarker of gut dysbiosis. J. Pers. Med..

[48] Cheng J., Hu J., Geng F., Nie S. (2022). Bacteroides utilization for dietary polysaccharides and their beneficial effects on gut health. Food Sci. Hum. Wellness.

[49] Tan H., Zhao J., Zhang H., Zhai Q., Chen W. (2019). Novel strains of *Bacteroides fragilis* and *Bacteroides ovatus* alleviate the LPS-induced inflammation in mice. Appl. Microbiol. Biotechnol..

[50] Ulsemer P., Henderson G., Toutounian K., Löffler A., Schmidt J., Karsten U., Blaut M., Goletz S. (2013). Specific humoral immune response to the Thomsen-Friedenreich tumor antigen (CD176) in mice after vaccination with the commensal bacterium *Bacteroides ovatus* D-6. Cancer Immunol. Immunother..

[51] Chen F., Zhang H., Zhao N., Yang X., Du E., Huang S., Guo W., Zhang W., Wei J. (2021). Effect of chlorogenic acid on intestinal inflammation, antioxidant status, and microbial community of young hens challenged with acute heat stress. Anim. Sci. J..

[52] Belkhou C., Tadeo R.T., Bacigalupe R., Valles-Colomer M., Chaffron S., Joossens M., Obregon A., Marin Reyes L., Trujillo O., Huys G.R.B. (2021). *Treponema peruense* sp. nov., a commensal spirochaete isolated from human faeces. Int. J. Syst. Evol. Microbiol..

[53] Demirkan I., Carter S., Winstanley C., Bruce K., McNair N., Woodside M., Hart C. (2001). Isolation and characterisation of a novel spirochaete from severe virulent ovine foot rot. J. Med. Microbiol..

[54] Zheng G., Wang D., Mao K., Wang M., Wang J., Xun W., Huang S. (2024). Exploring the Rumen Microbiota and Serum Metabolite Profile of Hainan Black Goats with Different Body Weights before Weaning. Animals.

[55] Seshadri R., Leahy S.C., Attwood G.T., Teh K.H., Lambie S.C., Cookson A.L., Eloe-Fadrosh E.A., Pavlopoulos G.A., Hadjithomas M., Varghese N.J. (2018). Cultivation and sequencing of rumen microbiome members from the Hungate1000 Collection. Nat. Biotechnol..

[56] Accetto T., Avguštin G. (2019). The diverse and extensive plant polysaccharide degradative apparatuses of the rumen and hindgut Prevotella species: A factor in their ubiquity?. Syst. Appl. Microbiol..

[57] Li C., Chen N., Zhang X., Shahzad K., Qi R., Zhang Z., Lu Z., Lu Y., Yu X., Zafar M.H. (2022). Mixed silage with Chinese cabbage waste enhances antioxidant ability by increasing ascorbate and aldarate metabolism through rumen *Prevotellaceae UCG-004* in Hu sheep. Front. Microbiol..

[58] Tang X., Zhang K., Xiong K. (2022). Fecal microbial changes in response to finishing pigs directly fed with fermented feed. Front. Vet. Sci..

[59] Jia Y., Shi Y., Qiao H. (2024). Bacterial community and diversity in the rumen of 11 Mongolian cattle as revealed by 16S rRNA amplicon sequencing. Sci. Rep..

[60] Sun B., Wang X., Bernstein S., Huffman M.A., Xia D.P., Gu Z., Chen R., Sheeran L.K., Wagner R.S., Li J. (2016). Marked variation between winter and spring gut microbiota in free-ranging Tibetan Macaques (*Macaca thibetana*). Sci. Rep..

[61] Garneau J.E., Tremblay D.M., Moineau S. (2008). Characterization of 1706, a virulent phage from *Lactococcus lactis* with similarities to prophages from other Firmicutes. Virology.

[62] Hallmaier-Wacker L.K., Lüert S., Gronow S., Spröer C., Overmann J., Buller N., Vaughan-Higgins R.J., Knauf S. (2019). A Metataxonomic Tool to Investigate the Diversity of *Treponema*. Front. Microbiol..

[63] Mølbak L., Klitgaard K., Jensen T.K., Fossi M., Boye M. (2006). Identification of a novel, invasive, not-yet-cultivated *Treponema* sp. in the large intestine of pigs by PCR amplification of the 16S rRNA gene. J. Clin. Microbiol..

[64] Wang J.G., Dou H.H., Liang Q.Y. (2025). Impact of Gut Microbiota and Inflammatory Cytokines on Immune Thrombocytopenia. Eur. J. Haematol..

[65] Andrade B.G., Bressani F.A., Cuadrat R.R., Tizioto P.C., de Oliveira P.S., Mourão G.B., Coutinho L.L., Reecy J.M., Koltes J.E., Walsh P. (2020). The structure of microbial populations in Nelore GIT reveals inter-dependency of methanogens in feces and rumen. J. Anim. Sci. Biotechnol..

[66] Wang Y., Zhang H., Zhu L., Xu Y., Liu N., Sun X., Hu L., Huang H., Wei K., Zhu R. (2018). Dynamic distribution of gut microbiota in goats at different ages and health states. Front. Microbiol..

[67] Lin Z.n., Ye L., Li Z.w., Huang X.s., Lu Z., Yang Y.q., Xing H.w., Bai J.y., Ying Z.y. (2020). Chinese herb feed additives improved the growth performance, meat quality, and nutrient digestibility parameters of pigs. Anim. Models Exp. Med..

[68] Zhou T., Zhang Z., Kim I. (2013). Effects of dietary *Coptis chinensis* herb extract on growth performance, nutrient digestibility, blood characteristics and meat quality in growing-finishing pigs. Asian-Australas. J. Anim. Sci..

[69] Yu Q.P., Feng D.Y., Xia M.H., He X.J., Liu Y.H., Tan H.Z., Zou S.G., Ou X.H., Zheng T., Cao Y. (2017). Effects of a traditional Chinese medicine formula supplementation on growth performance, carcass characteristics, meat quality and fatty acid profiles of finishing pigs. Livest. Sci..

